# House modifications using insecticide-treated screening of eave and window as a vector control tool: evidence from a semi-field system in Tanzania and simulated epidemiological impact

**DOI:** 10.1186/s12889-026-28554-z

**Published:** 2026-07-17

**Authors:** Olukayode G. Odufuwa, Richard J. Sheppard, Safina Ngonyani, Ahmadi Bakari Mpelepele, Dickson Kobe, Agathus Njohole, Jason Moore, Jastin Lusoli Lusoli, Joseph B. Muganga, Rune Bosselmann, Ole Skovmand, Zawadi Mageni Mboma, Emmanuel Mbuba, Rose Philipo, Jennifer C Stevenson, Ellie Sherrard-Smith, John Bradley, Sarah Jane Moore

**Affiliations:** 1 Vector Control Product Testing Unit (VCPTU) Ifakara Health Institute, Environmental Health, and Ecological Sciences, P.O. Box 74, Bagamoyo, Tanzania; 2 Vector Biology Unit, Department of Epidemiology and Public Health, Swiss Tropical & Public Health Institute, Kreuzstrasse 2, Allschwill, 4123 Switzerland; 3 Faculty of Science, University of Basel, Petersplatz 1, Basel, 4001 Switzerland; 4 MRC International Statistics and Epidemiology Group, London School of Hygiene and Tropical Medicine (LSHTM), London, WC1E 7HT UK; 5 MRC Centre for Global Infectious Disease Analysis, Department of Infectious Disease Epidemiology, Imperial College, London, United Kingdom; 6 Vegro Aps, Copenhagen, Denmark; 7 VectorisIQ SARL, Montpellier, France; 8 School of Life Sciences and Bioengineering, The Nelson Mandela African Institution of Science and Technology (NM-AIST), P.O. Box 447, Arusha, Tanzania

**Keywords:** Insecticide-treated screening (ITS), Insecticide-treated bednets (ITNs), Malaria, Dengue, Nuisance biting, Semi-field system, Pyrethroid, Piperonyl butoxide (PBO), Tanzania, Transmission modelling

## Abstract

**Background:**

Gaps in unimproved house structures, especially in eaves and windows, allow mosquito entry, increasing indoor vector-borne disease transmission. Simple modifications to such houses may reduce human exposure, and insecticide treatment may kill mosquitoes, benefiting all community members. This study evaluated insecticide-treated screening (ITS) for eaves and windows, incorporated with deltamethrin and piperonyl butoxide (PBO), compared to a permethrin and PBO-treated bednet in Tanzania.

**Method:**

A randomised Latin-square design (4 × 4) was used in four experimental huts within a large netting cage to allow mosquito recapture inside and outside of huts. Four treatments were evaluated: (1) new (12-month stored) ITS; (2) 12-month naturally-aged ITS; (3) 12-month field-used pyrethroid-PBO bednet (standard of care in Tanzania), and (4) no treatment. The study was performed for 32 nights using 30 mosquitoes per strain, per hut per night. Four laboratory-reared strains were used: malaria vectors (*Anopheles arabiensis* and *An. funestus*), dengue vector (*Aedes aegypti*), and nuisance biting (*Culex quinquefasciatus*). Recaptured mosquitoes were assessed for mortality at 72 h, blood-feeding, and hut entry. A simulation with a modified mechanistic model tracking *Plasmodium falciparum* malaria was used to illustrate potential epidemiological impact from these products.

**Results:**

Against all mosquito species compared to 12-month aged pyrethroid-PBO-treated bednet, new ITS induced higher mortality [Odds Ratio:2.25(95%Confidence Interval:1.65–3.06),*p* < 0.0001], and aged-ITS was similar [OR:0.80(95%CI:0.59–1.08),*p* = 0.141]. Both new and aged ITS significantly (*p* < 0.0001) reduced mosquito blood-feeding [new OR:0.02(95% CI:0.01–0.03); aged OR:0.09(95%CI:0.05–0.14)] and hut entry [new IRR:0.10(95%CI:0.08–0.13); aged IRR:0.25(95%CI:0.21–0.31)]. Transmission model estimates indicate epidemiological impacts of ITS may supersede pyrethroid-PBO-treated bednets at the population level. The model results indicate impact potency depends on assumed intervention percentage cover, durability, and mosquito bionomics.

**Conclusions:**

This study introduces a standardised semi-field bioassay to evaluate ITS, generating entomological data that, for the first time, enabled modelling of its potential impact on malaria transmission. ITS is an efficacious tool for public health as it kills substantial proportions of malaria and dengue vectors, and reduces nuisance biting. Given its simplicity, it should be considered an additional or stand-alone tool for screening unimproved houses.

**Supplementary Information:**

The online version contains supplementary material available at https://doi.org/10.1186/s12889-026-28554-z.

## Background

Insecticide-treated bednets (ITNs) and indoor residual spraying (IRS) are widely used and have contributed to the majority (> 80%) [1] of the 2.1 billion malaria cases and 11.7 million malaria deaths averted globally in the period 2000–2020 [2]. ITNs are hung over sleeping spaces, thereby providing an insecticidal barrier between the user and mosquitoes at night [3], while IRS applies insecticide to surfaces where mosquitoes rest [4]. The insecticides used in public health are generally contact toxicants that induce mortality, while some are also contact irritants, resulting in reduced mosquito blood-feeding; they may also disable mosquitoes, causing a reduction in blood-feeding, disorientation, or impeding mosquito reproduction [5]. When these interventions are used by the majority (around 80% [6]) of the community, disease reduction among all community members, even non-users, is often observed [7–12], due to the impact the interventions have on the mosquito population as a whole. The widespread use of malaria control interventions can also impact other mosquitoes, including those transmitting dengue [13] and those that are nuisance-biting; ITN adherence and IRS acceptance is often improved when nuisance-biting insects are also reduced [14, 15]. Despite decades of widespread use of ITNs and IRS in sub-Saharan Africa, vector-borne diseases, in particular malaria and dengue, have been increasing or stalling since the year 2020 [2, 16, 17], indicating that additional effort is needed for more effective control of these diseases.

Reviews of studies on house screening [18, 19] and a trial of a lethal house lure (insecticidal tubes installed in the eaves of houses) [20] conducted in a range of African settings report the positive effect of house modification for malaria control. Based on the proven efficacy against malaria, house screening is recommended as a supplementary tool to ITNs or IRS by the World Health Organization (WHO) [11]. Properly sealing mosquito entry points of housing structures such as eaves, windows, doors, ceilings, and traditional walls or using insecticide-incorporated materials for house construction reduces indoor mosquito abundance and therefore vector exposure of all household members [18] indoors and outside of sleeping hours [21, 22]. Many houses in Africa, especially those in rural areas are unimproved (houses with walls, roofs, and floors built with natural or rudimentary materials including mud, thatch, wood, bamboo, earth, sand [18]). The majority of these houses have open eaves and windows, and such potential mosquito entry points may be essential for indoor cooling and entry of natural light [23]. Simple closure of eaves can reduce air circulation and has the potential to increase indoor temperature, so it is often not acceptable in tropical regions [24]. Although there are existing house modification tools, many of them are unsuitable for unimproved houses [25, 26] and are expensive to install.

Insecticide-treated screening (ITS) of eave entry points and windows [27] may be used on unimproved houses. In a pilot study conducted in western Kenya [28], the netting used on these entry points was incorporated with a pyrethroid insecticide and proved to be efficacious at reducing mosquito indoor densities, and was cost-effective and acceptable to users. In areas with pyrethroid-resistant mosquitoes, pyrethroid-only materials may not be fully efficacious. Using ITS incorporated with pyrethroids and piperonyl butoxide (PBO) - a synergist used to restore the susceptibility of mosquitoes with metabolic resistance mechanisms to pyrethroids - may have a greater impact against resistant mosquitoes [29].

Field-based experimental hut trials have been used for assessing the efficacy of indoor interventions, including ITNs and IRS [30, 31]. The entomological efficacy estimates are reliably assessed based on recaptured mosquitoes from inside the hut (or from within exit traps) [30]. However, the mortality induced by such interventions cannot be measured using experimental huts as dead mosquitoes cannot be recovered in an open setting. To assess insecticidal housing modification including ITS, it is more appropriate to use a semi-field system (SFS) with huts built inside a netting cage [32, 33]. This is to allow the recapture of mosquitoes for the assessment of delayed mortality, the primary endpoint considered by WHO for evaluation of fast-active neurotoxic insecticides incorporated vector control tools [34]. Results from studies such as this have been used previously to parameterise transmission models that can extrapolate the potential impact of interventions for community protection over a range of socioecological settings [35]. We are unaware of work to explicitly model house screening, however, eave tubes have been simulated previously [36].

In this study, ITS treated with pyrethroid-PBO were evaluated for their entomological efficacy against laboratory-reared pyrethroid-resistant malaria and dengue vectors, and nuisance biters in a SFS located in Tanzania, given that the tool has the potential to control populations of all these mosquitoes [37–39]. In addition, we extended an established *Plasmodium falciparum* transmission model [35–37] to replicate the installation of treated screens, simulate the impact of bednets and screening on malaria prevalence and incidence, and theorise on the potential epidemiological efficacy of these products.

## Methods

### Semi-field study location

**Fig. 1:**
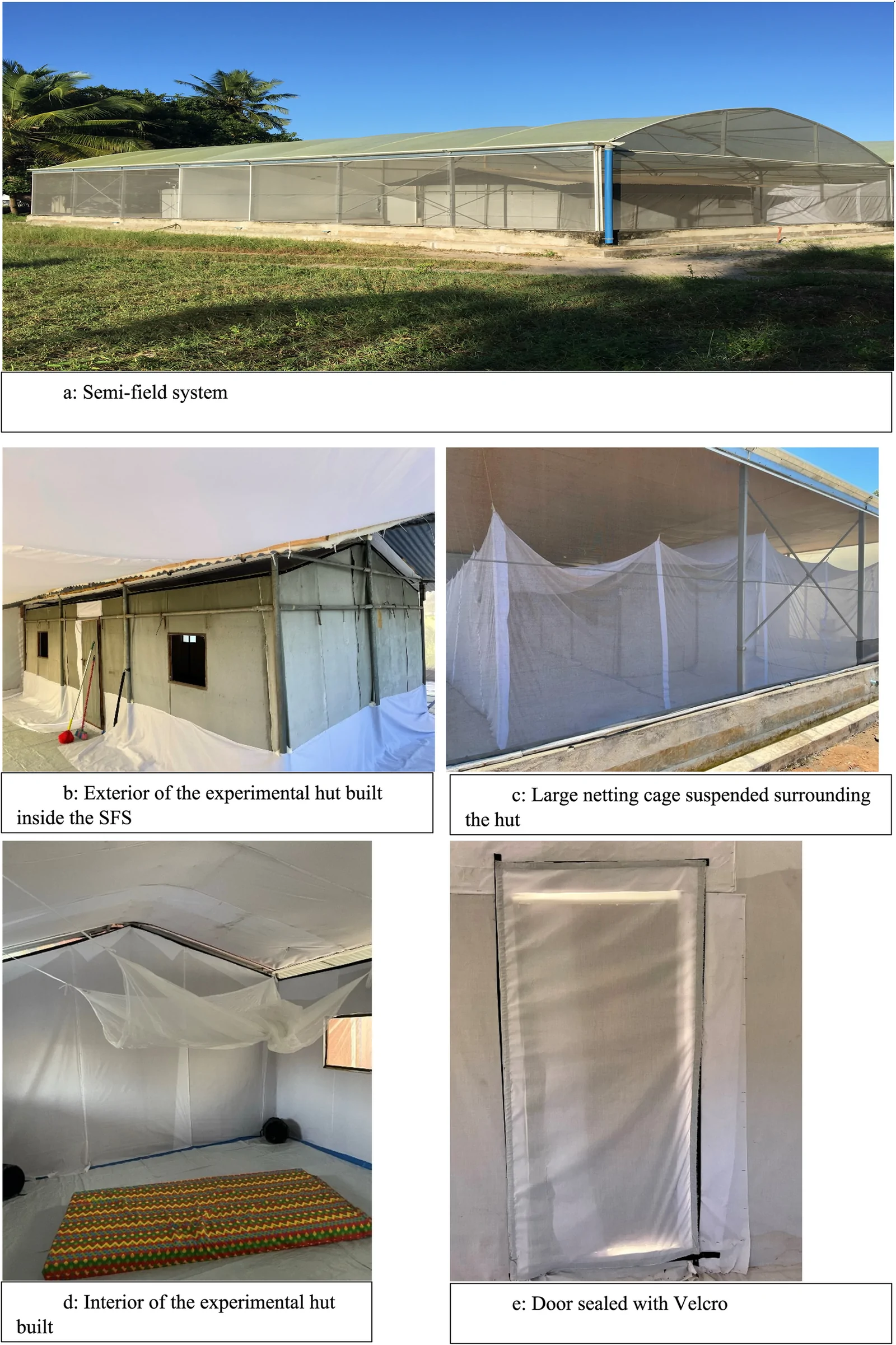
The semi-field system at Bagamoyo, Tanzania

The trial was conducted in the semi-field system (SFS) (Fig. 1), of the Ifakara Health Institute in Bagamoyo, Tanzania (6°25’42.4"S 38°53’01.9"E, Figure S1). The system measures 28.8 m (m) x 21 m and is built on a raised concrete slab with a water-filled channel to prevent entry of scavenging ants. The external walls are made of untreated durable-shade netting and the roof is from polyethylene sheeting (Fig. 1a). The inside of the SFS is divided into four chambers, with an internal second large double-layered cotton-netting cage (9 m width x 8.5 m length x 2.7 m height) suspended in each chamber. Inside this cage, an Ifakara experimental hut was constructed and used without window traps or eave baffles [40] (Fig. 1b). In the inner cage (Fig. 1c), white cloth-lining is laid on the floor in the hut and 12 resting buckets (Fig. 1d) were used to maximise mosquito recapture. One bucket was placed at each corner inside the experimental hut, one at each corner of the chamber, and one at each side of the experimental hut. White, heavy-duty cotton was used to cover the door of the experimental hut and attached using Velcro tape (Fig. 1e).

### Study design

A randomised Latin square (4 × 4) design [41] was carried out to compare the efficacy of (1) new (one-year stored in a non-air-conditioned, standard 20-foot metal shipping container covered with a roof and heavy-duty aluminium foil as the ceiling) insecticide-treated screening (ITS, co-installation on eaves and windows), (2) 12-month naturally-aged ITS, (3) 12-month naturally-aged Olyset^®^ Plus insecticide-treated bednet (ITN) and (4) no treatment. Each arm and volunteer was randomly allocated to one of the four chambers on the first night of the test, followed by a sequential nightly rotation of volunteers and rotation of arms at the end of every four nights. The test was conducted over 32 experimental nights using four strains of laboratory-reared mosquitoes. Quality checks were conducted on net samples (25 centimetre (cm) x 25 cm) using World Health Organization (WHO) cone bioassays to determine bioefficacy and using the Collaborative International Pesticides Analytical Council (331/LN/M/3) method to assess insecticide content, before and after the trial [30].

### Description of test items

The material of ITS for house screening [28] was a net consisting of two 0.152 mm (mm) diameter and 140-denier polyethylene yarns: the first yarn was incorporated with deltamethrin plus piperonyl butoxide (PBO) and the second yarn with PBO only. The two yarns were knitted together to provide a net with 120 milligrams per metre square (mg/m²) of deltamethrin and 540 mg/m² PBO. The nets were manufactured in August, 2020 by Vegro Aps, Denmark, and delivered to existing IHI storage (non-air-conditioned, standard 20-foot metal shipping container covered with a roof and heavy-duty aluminium foil as the ceiling) at that time in April 2021. Incorporating the PBO synergist with pyrethroid aimed to control those mosquitoes with resistance to pyrethroids due to the upregulation of mixed function oxidases [29]. Olyset^®^ Plus ITN was selected as the positive comparator being the first in class pyrethroid-PBO ITN [42], which is recommended by WHO for malaria control against pyrethroid-resistant mosquitoes [43, 44], and it is the standard of care in Tanzania [45]. The Olyset^®^ Plus ITN is a 150-denier knitted monofilament polyethylene yarn incorporated with 20 g of active ingredient per kilogram (g AI/kg), which is about 800 mg of AI/m^2^) permethrin and 10 g/kg (about 400 mg/m^2^) PBO, manufactured by Sumitomo Chemical Co., Limited, Japan. A hut with no ITN and no screening was included. This “no treatment” arm also acted as the control comparator arm for the modelling simulations reported below.

### Preparation of treatments for natural ageing, washing, and deliberate holing

**Fig. 2:**
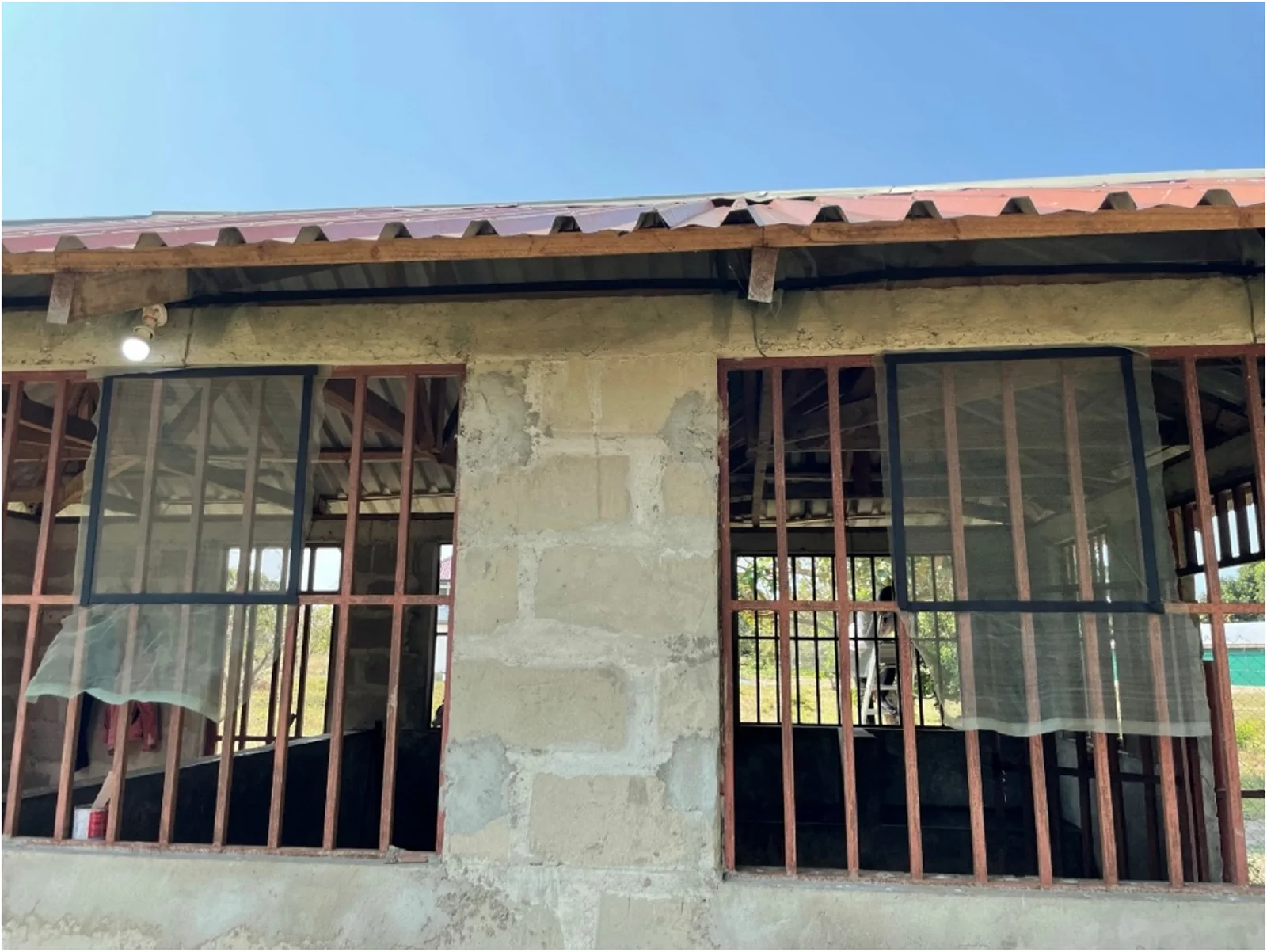
Natural ageing of insecticide-treated screening

In August 2021, using Velcro tape, ITS measuring approximately 0.5 m x 23 m were installed on the eaves, and pieces measuring 1 m x 0.7 m on each of the four windows of an animal house located at the site of the Ifakara Health Institute in Bagamoyo (Fig. 2). They were left to naturally age until the end of September 2022. The remaining of the screening material was returned to the IHI storage (Regarded as new ITS in this study).

**Fig. 3:**
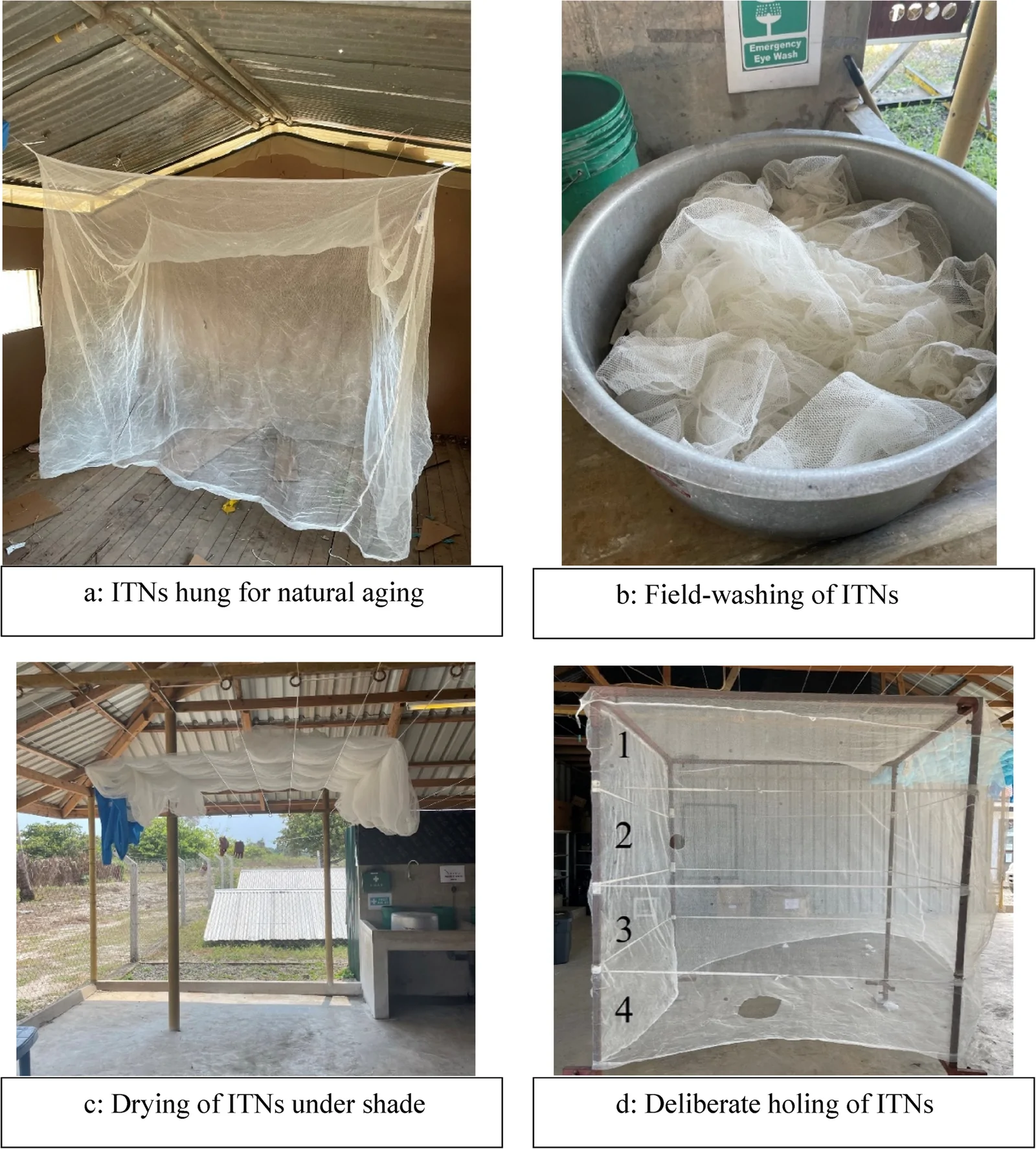
Natural ageing of Olyset^®^ Plus insecticide-treated bednets

On the same day, two new Olyset^®^ Plus ITNs (one for the experiment and one spare) were hung inside an experimental hut (Fig. 3a), located 60 m from the animal house where ITS was installed. Based on data from a community trial indicating an average of six washes per year of Olyset^®^ Plus ITNs in Tanzania [46], ITNs were periodically field-washed following WHO guidelines [47] in October and December 2021, as well as January, June, July and August 2022. Each of the ITNs was soap-washed once using two grams/litre of soap (“Jamaa palm oil” soap flakes) and water-rinsed twice in an aluminium bowl filled with 10 L of filtered well water (maximum hardness of five dH) (Fig. 3b). Each wash or rinse was standardised, stirring the bednet with a gloved hand at 20 rotations per minute for three minutes, soaking for four minutes, and stirring again for three minutes. ITNs were dried horizontally in the shade where the conditions were no higher than 32 degrees centigrade (°C), and no direct sunlight (Fig. 3c). After each wash and drying, ITNs were returned into the experimental hut for continued natural aging (Fig. 3a).

Shortly before testing aged Olyset^®^ Plus ITN in the SFS, the ITNs were deliberately holed to mimic field-use damage following estimated sizes and number of holes reported for one-year-old Olyset^®^ Plus ITNs tested under field conditions in Tanzania [46] (Fig. 3d) (Table 1). Holes were equally spread across the four sides of the ITNs.

**Table 1:** The size and number of holes made on study Olyset^®^ Plus insecticide-treated bednets to simulate user condition

Size	Zone 1 (upper strip)	Zone 2 (below upper strip)	Zone 3 (above bottom strip)	Zone 4 (bottom strip)	Roof
Number of holes and Estimated size	28 x size 1 (1.25 cm)	5 x size 1 (1.25 cm) 1 x size 2 (6 cm)	14 x size 1 (1.25 cm) 2 x size 2 (6 cm)	15 x size 1 (1.25 cm) 4 x size 2 (6 cm) 1 x size 3 (17.5 cm)	24 x size 1 (1.25 cm) 5 x size 2 (6 cm) 2 x size 3 (17.5 cm)

### Description of study mosquitoes

Four laboratory-reared strains of mosquitoes were used: *Anopheles (An.) arabiensis* (Kingani strain), *An. funestus* (FUMOZ strain), *Aedes (Ae.) aegypti* (Bagamoyo strain), and *Culex (Cx.) quinquefasciatus* (Bagamoyo strain). Major malaria vectors in Tanzania are *An. arabiensis* and *An. funestus* [48, 49], *Ae. aegypti* transmits dengue [50] and *Cx. quinquefasciatus* is notable for nuisance biting [51]. *Ae. aegypti* was pyrethroid-susceptible, while the other three strains were resistant to pyrethroids, with susceptibility being fully or partially restored through pre-exposure to PBO (Table S1).

The colonies are maintained by feeding *Anopheles* larvae on Tetramin fish food, *Aedes* and *Culex* on cat biscuits (Purina) and adults on cattle blood via membrane between three and six days after emergence and 10% sugar solution *ad libitum*. Temperature and relative humidity within the insectary are maintained between 27 ± 2 °C and 50–99% following MR4 guidance [52].

Mosquitoes released in the SFS chambers were non-blood-fed females, aged between five and eight days, sugar-starved for six hours before release. Mosquitoes exposed to cone bioassay for quality checks before and after the SFS were nulliparous sugar-fed and aged between three to five days.

### Semi-field system experiment procedure

Testing in the SFS commenced in October 2022. Four male adults (≥ 18 years old) experienced in mosquito collection from the villages around the Ifakara Health Institute site of Bagamoyo were recruited following written informed consent for their participation in the study. Volunteers were trained in mosquito collection.

For each experimental day, chambers were cleaned and damp cotton towels were placed in resting buckets to provide a dark humid refuge for mosquitoes. Each volunteer was provided with four netted paper cups containing 30 mosquitoes per strain, mouth aspirators, and mechanical Prokopack aspirators. Mosquitoes were allowed to acclimatise to the SFS for 30 min before release. The inner net chamber was closed, and at 18:00 hours (h), each volunteer released the mosquitoes from the paper cups at a fixed point behind the experimental hut, allowing mosquitoes to disperse and behave as naturally as possible. Volunteers directly entered the experimental hut, shut, and sealed the door with white cotton (Fig. 1e). Each volunteer slept overnight in the hut until 07:00 h.

From 07:00 h, mosquitoes were collected by the volunteers using both mouth aspirators and a mechanical Prokopack, collecting mosquitoes from within the hut and then from outside the hut within the chambers. Additional checking for remaining mosquitoes was done by the supervisors. Cups were labelled with the location of collection, inside or outside of the hut, then transported to the laboratory. Mosquitoes were sorted and scored by location (inside or outside hut) as dead and fed, dead and unfed, alive and fed, or alive and unfed. Mosquito survivors were provided 10 percent (%) sugar solution and held for 72 h in a temperature and relative humidity-controlled room to record mortality at 72 h post-exposure.

In the first four days of the trial, a short questionnaire was administered to the participants that slept in the hut to record perceived adverse effects.

### Quality checks of samples from insecticide-treated screening and Olyset^®^ plus insecticide-treated bednets

Quality checks were conducted to verify the efficacy of new ITS and Olyset^®^ Plus ITN. A sample (25 cm x 25 cm) was removed from the centre of each of the three rolls of the treated screening material, 1.0 m from the end of the roll, given that the surface might have been exposed to unsuitable conditions during shipment. Three net sections were cut from standardised locations of one new Olyset^®^ Plus ITN from the same batch as that tested in the SFS evaluation, and were performed in June 2021.

After the SFS evaluation, additional cone tests were conducted in January 2023 to assess the impact of artificial ageing on the treated materials. Samples (25 cm x 25 cm) were removed from the eaves and windows of the new and aged materials (Figure S2) and five samples were removed from standardised locations of the aged Olyset^®^ Plus ITN.

All netting samples were tested against the four laboratory strains used in the SFS experiments using cone bioassays. For each assay, four replicates with five mosquitoes per replicate (20 mosquitoes used per sample) were tested following the 2013 WHO guidelines [47]. Mortality was observed after 24 h for quality checks performed before SFS testing, and 72 h for post SFS based on evidence of increased mortality at 72 h for metabolic-resistant mosquitoes [53]. Four samples (Figure S2, E1, E2, W1, and W2) were randomly selected for chemical analysis.

### Data Management and analysis

Data were recorded on paper forms and double-entered in Microsoft^®^ Excel^®^ (Microsoft Corporation, 2018). The two entries were then merged to verify the accuracy of the data before analysis in Stata 16 (Stata Corp, Texas, USA). Data was cleaned by checking the balance of treatments, chambers, volunteers, and mosquito strains. Number of mosquitoes recaptured at the end of the exposure period (12 h after release) and then assessed 72 h later) were verified against the number of mosquitoes released. Where discrepancies were found, paper forms were checked and corrections were made accordingly.

From the mosquitoes collected, mosquito mortality at 72 h (unfed and dead plus fed and dead scored 72 h after recapture, M72), blood-fed (fed and alive plus fed and dead scored immediately after collection) and deterred (all mosquitoes collected inside the hut scored immediately after collection) were calculated for each arm. Descriptive statistics presented percentage arithmetic means with 95% confidence intervals of endpoints (M72 & blood-feeding for SFS, and knockdown and M72 for cone bioassay). The median and interquartile range (IQR) of the number of mosquitoes captured inside the hut were presented. For cone bioassays, mortality in the treated netting arms was adjusted by the mortality in the untreated control arm using Abbott’s formula because mortality in the untreated net was 5% [54].

Mixed effects binomial logistic regression was used to evaluate differences between arms for the M72 and blood-feeding endpoints. A negative binomial mixed effects model was performed to compare the total number of mosquitoes recaptured inside the hut between arms to compare deterrence. Hut position (location of hut within the SFS to account for positional effects), sleeper, and day were included as factor-fixed effects, and observations (each row of data) as random effects to account for overall variation in the study.

### Transmission modelling to investigate potential epidemiological impact at the population level

A transmission model is used to explore how the use of ITS might contribute to the control of *falciparum* malaria. The model that we use [55] simulates the transmission of *falciparum* malaria parasites between humans and *Anopheles* mosquitoes. The model has been fitted previously to data from across malaria-endemic Africa to capture how immunity, seasonal mosquito densities, local demography, and the use of other interventions alter transmission to give us a tool that can reliably recreate the endemicity that is witnessed in sub-Saharan Africa [56]. We have also validated the use of ITNs by testing the capacity of parameters defining impact to recreate the prevalence from cluster-randomised trials in model simulations [57]. In this contribution, we add the capacity to simulate the ITS as an intervention that inhibits mosquitoes from entering a house, with the potential to force a proportion attempting to feed on humans indoors to repeat their foraging cycle, and that can induce mosquito mortality for a proportion of mosquitoes attempting to feed indoors. The full details of the transmission model changes are relayed in Supplementary Material 1.

In this contribution, the SFS data are used to quantify the effect from the ITS. In previous work, meta-analyses of experimental hut trials have been used to quantify the entomological impact of ITNs by taking the estimates for mosquitoes that are killed, fed, or deterred (the numbers in the hut) in a given treated hut, and weighting these estimates by the deterrence estimate that is a comparison between mosquitoes caught within the untreated control hut and the treated hut [58] to generate parameter values for the model [57, 59]. Combining the data in this way allows an estimate of the model parameter values for the probabilities of mosquitoes successfully feeding, being forced to repeat the foraging attempt, or being killed when an ITN is present, and works optimally (i.e. indicating the potency when the bednet is new) [57, 59, 60]. These methods can be adapted to the ITS, but the deaths from screens occurs twice, for entry and exit, and we assume that mosquitos that do not exit eventually die.

The SFS data (see Supplementary Material 2) is used here to parameterise the ITN and ITS impacts within the model simulations (details provided in Supplementary Material 1). However, the aged ITN provides a measure of the impact at one year and to appropriately simulate the effects, we need to translate this to an optimal impact i.e. the impact that would be afforded on first deploying new ITNs to the community.

The full durability of ITNs is unknown, so – for modelling purposes –an assumption is made on how rapidly these impacts decay following the mass campaign. Previously, a mean duration of mortality-inducing impact has been estimated by Griffin et al. (2010) [69] from Mahama et al. (2007) [73] for pyrethroid-only ITNs using washed ITNs, and the same logic applied for pyrethroid-PBO ITNs [59]. This assumes that the killing effect from ITNs will decrease at a constant rate.

In the absence of further information on the durability of the ITS, we explore a reasonable range in the mean duration of the product mortality-inducing impact that would return mortality estimates after 365 days within the range of the SFS results.

In addition, we include simulations that use pyrethroid-PBO ITNs parameters developed via the statistical framework and validated against gold-standard randomised control trial data for testing the epidemiological impact of these interventions [57]. In the review work preceding the validation exercise [58], associations between entomological impact and the level of phenotypic resistance in *Anopheles* populations were statistically determined allowing the resistance-level specific parameterisation of impacts on mosquitoes from ITNs within the transmission model. Values aligning with the resistance status of the species *An. arabiensis* and *An. funestus* tested within the SFS are used here for the model simulation exercise (See Table S2, and Supplementary Material 1).

### Scenario analysis

We use the adjusted transmission model to simulate a set of theoretical intervention scenarios. To manage feasibility in the number of simulations run, and to specifically explore the comparison between pyrethroid-PBO ITNs and ITS, for all simulations, we use the median model parameters that have been fitted previously for malaria simulation [55, 60–62], except for the values indicated in Table S2. This is a limitation as we do not express full uncertainty, but this serves our purpose of illustrating the potential impact from ITS and the comparable impact with pyrethroid-PBO ITNs. For the setup, we use a theoretical setting with malaria prevalence in children of 6 to 59 months of age reaching about 60% during the peak season, with a single transmission season profile. In all simulations, treatment of clinically ill patients is implemented so that 45% of people receive artemisinin combination therapy (ACT) when needed, and a further 15% receive non-ACT treatment. It is assumed that 50% of mosquitoes have the *An. arabiensis-*like bionomics and 50% have the *An. funestus-*like bionomics (Table S3) and corresponding impacts from the ITN or ITS interventions (Table S2) given the range in resistance expressed during tube bioassay testing (Table S1) as determined by Nash et al. [58] and validated in Sherrard-Smith et al. [57]. Adherence to ITN use is also simulated to wane over time [63] so that by three years, half as many people are using the original ITN. Each simulation is run for 15 years. The first six years (year 0) generate a relative equilibrium before we switch to comparative scenarios. Pyrethroid-PBO ITNs are implemented every three years from the start, depending on the scenario explored, the ITS is also implemented on three-year cycles from year 0. It is assumed that ITN and ITS interventions are deployed overnight and to the target proportion of the population, who immediately replace old nets with the newly distributed ones and gain the benefit from their impact. In reality, it will take longer to provide a community with ITS, but the longer-term gains should level out for this theoretical exploration of epidemiological impacts.

To consider the potential impact, of each intervention class (pyrethroid-PBO ITNs or ITS), a counterfactual simulation is also run where ITNs are distributed in years − 6 and − 3 but then no longer deployed. Older ITNs may still circulate in the population but will have a far lower efficacy given that insecticide would have waned, and use fallen to a low level. To estimate reductions in prevalence, the average prevalence in children of 6–59 months of age, 5–15 years, and for all ages, over years 0–3 for the treatment simulation (*C*_*T*_) are compared to the corresponding estimate from the counterfactual simulation (*C*_*C*_). To estimate cases averted, the same age groups are considered across three years to generate an estimated number of cases averted per 1,000 population. That is:

$$\:Epidemiological\:Impact=\:100\:\times\:\frac{\left({C}_{C}-{C}_{T}\right)}{{C}_{c}}$$

Where *C* can indicate prevalence or cases as required.

The pyrethroid-PBO ITNs – parameterised using the meta-analysis approach [57, 58] or using the directly comparable approach by generating parameters from the SFS data, and ITS products are simulated as stand-alone or combined interventions. Code is provided as Supplementary Material 3.

Next, to consider the potential of this novel product, a simple sensitivity analysis is performed on the population ITS coverage and product durability (the mean duration of the mortality-inducing impact). The counterfactual is the use of pyrethroid-PBO ITNs, parameterised as per the meta-analysis [57, 58], distributed every three years to 60% of the population on deployment. Here, we simulate the median estimated impact from the ITS given increasing cover from 0 to 80% of the community, and for a product that has a mean duration of one year through to five years.

## Results

### Experimental validity

Throughout the whole year of natural aging in the experimental hut, the median temperature was 27.2 °C [range (R): 17.4–47.4 °C] and relative humidity was 82.0% (R: 0–100%).

Nightly (6:00 PM to 7:00 AM), the median temperature and relative humidity in chambers outside of the experimental huts were 26.4 °C (R: 20.6–29.5) and 91.4% (R: 67.5–100), respectively. The median indoor temperature of huts installed with ITS was 25.2 °C (R: 18.3–30.6), and this was similar to huts with Olyset^®^ Plus ITN 25.2 °C (R: 20.7–26.2), and huts with no treatment 25.0 °C (R: 17.8–28.3), indicating that ITS did not increase indoor temperature (P-value = 0.1476 estimated using the Two-sample Wilcoxon rank-sum (Mann-Whitney) test).

Recaptured mosquitoes from the chambers of the SFS were held at a median temperature of 25.6 °C (R: 22.6–27.8) and relative humidity of 78.3% (R:48.8–89.3) to assess M72, with 1% and 5% were out of WHO thresholds for temperature (27 ± 2 °C) and relative humidity (80 ± 20%) respectively, for laboratory testing [30].

Overall, 85% (13,370 of 15,722) of the mosquitoes released were recaptured in this study: 85% (3,300 of 3,883) of *Anopheles (An.) arabiensis*, 80% (3,186 of 3,940) of *An. funestus*, 87% (3,457 of 3,970) of *Aedes (Ae.) aegypti* and 87% (3,427 of 3,929) of *Culex (Cx.) quinquefasciatus*, mosquitoes. The proportion of overall mosquitoes recaptured varied slightly among the arms with more mosquitoes recaptured in the untreated arm (93%) than the aged Olyset^®^ Plus net (81%), new ITS (78%), and aged ITS (87%) (Table S4).

### Insecticide-treated screening has the potential to provide a community effect against mosquito vectors

**Fig. 4:**
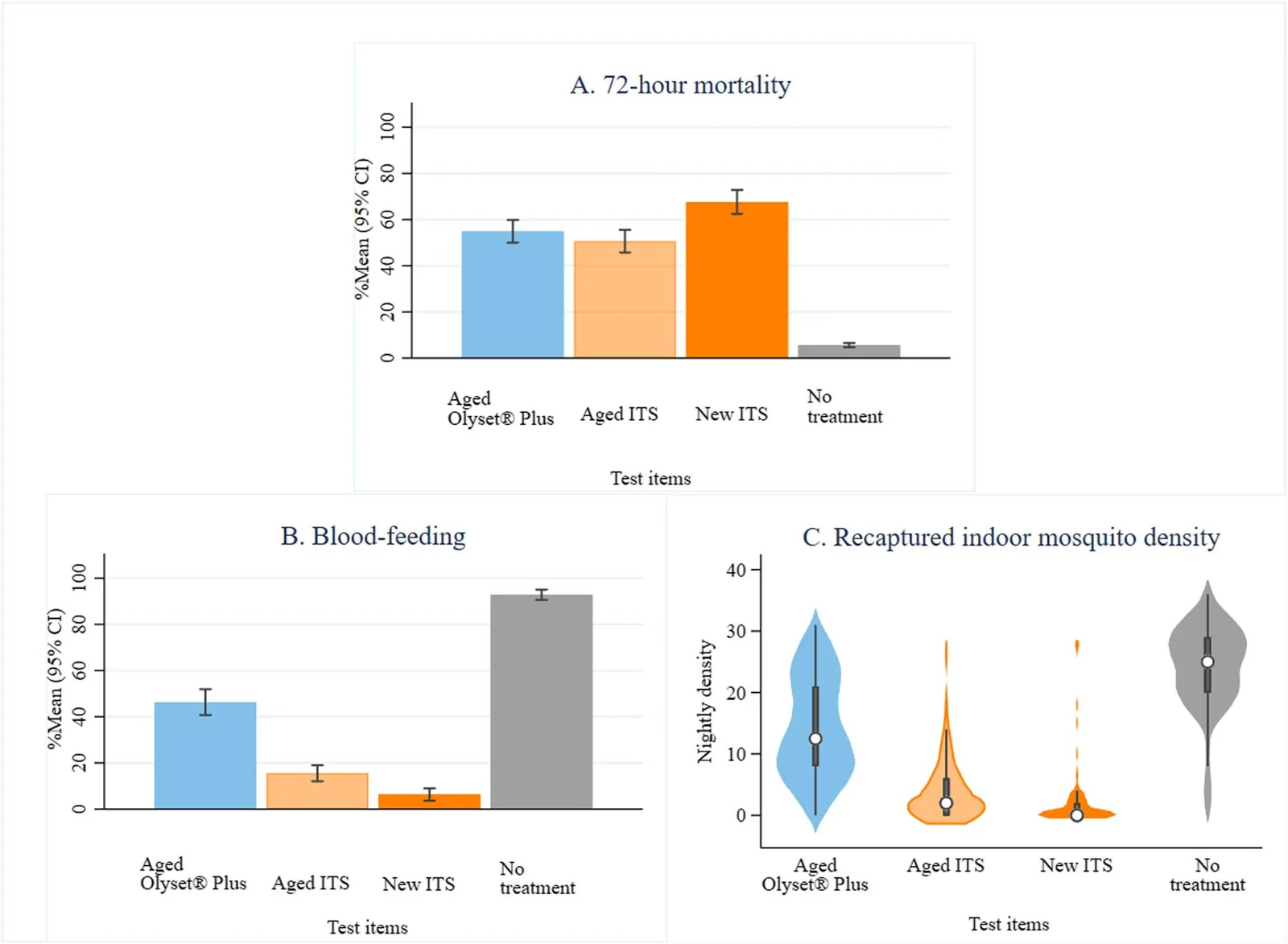
The proportion of mortality (**A**) and blood-feeding (**B**), and median indoors of overall mosquitoes (pyrethroid-resistant *An. arabiensis*, *An. funestus*,* Cx. quinquefasciatus* and susceptible *Ae. aegypti*) recaptured after exposure to experimental huts installed with insecticide-treated screen against the standard of care: Olyset^®^ Plus bednet in the semi-field system in Tanzania

ITS induced mortality to mosquitoes measured at 72 h (M72). Overall (all strains combined), the M72 among mosquitoes exposed to the aged ITS were similar to those exposed to the aged Olyset^®^ Plus ITN [51% vs. 56%, Odds Ratio (OR): 0.80 (95% Confidence Interval: 0.59–1.08), p-value = 0.141], whereas the M72 was significantly higher for the new ITS in comparison to aged Olyset^®^ Plus net [68% vs. 55%, OR: 2.25 (95% CI: 1.65–3.06), p-value < 0.0001] (Table 2; Fig. 4).

**Table 2:** Mortality at 72 h, blood-feeding, and deterrence of mosquitoes exposed to insecticide-treated screening compared to a standard of care pyrethroid-PBO insecticide-treated bednet, Olyset^®^ Plus in the semi-field system. All mosquito strains combined

Test items	Total recaptured (*N*)	Total (*n*)	%Arithmetic mean (95% CI)	OR (95% CI)	*P*-value
72-hour Mortality					
Aged Olyset^®^ Plus	3,209	1,748	54.9 (50.0–59.8)	1.00	
Aged ITS	3,424	1,670	50.6 (45.7–55.5)	0.80 (0.59–1.08)	0.141
New ITS	3,027	2,002	67.6 (62.4–72.8)	2.25 (1.65–3.06)	< 0.0001
* No treatment*	3,710	204	5.6 (4.7–6.5)	0.03 (0.02–0.04)	< 0.0001
Blood-feeding					
Aged Olyset^®^ Plus		1,537	46.3 (40.7–51.9)	1.00	
Aged ITS		511	15.5 (12.0–19.0)	0.09 (0.05–0.14)	< 0.0001
New ITS		178	6.3 (3.7–9.0)	0.02 (0.01–0.03)	< 0.0001
* No treatment*		3,447	92.8 (90.6–95.0)	66.33 (39.50–111.38)	< 0.0001
Indoor density			Median (IQR)	IRR (95% CI)	
Aged Olyset^®^ Plus		1,832	13 (8–22)	1.00	
Aged ITS		583	2 (0–6)	0.25 (0.21–0.31)	< 0.0001
New ITS		271	0 (0–2)	0.10 (0.08–0.13)	< 0.0001
* No treatment*		3,022	25 (20–29)	1.73 (1.46–2.06)	< 0.0001

Treatment, volunteer, hut, day, and species are factor-fixed effects, and observation was a random effect.

*OR* Odds ratio, *IQR* Interquartile range, *IRR* Incidence rate ratio, *CI* Confidence Intervals

There was variation in the M72 observed within the strains (Table S5). While *An. arabiensis*, *Cx. quinquefasciatus* and *Ae. aegypti* mosquitoes tested against aged ITS died in similar proportions to those exposed to aged Olyset^®^ Plus net (*p* > 0.05), a significantly lower M72 was observed for *An. funestus* mosquitoes exposed to the aged ITS arm in comparison to the aged Olyset^®^ Plus net [66% vs. 79%, OR: 0.44 (95% CI: 0.27–0.72), *p* = 0.001]. For the new ITS, significantly higher proportions of *An. arabiensis* (p = < 0.0001), *An. funestus* (*p* = 0.002) and *Ae. aegypti* (p = < 0.0001) mosquitoes died at 72 h after exposure to ITS compared to those exposed to the aged Olyset^®^ plus ITN, however no significant difference was found for *Cx. quinquefasciatus* mosquitoes. All treatments had higher mortality than the untreated arm (Table S5).

### Insecticide-treated screening reduces blood-feeding

Overall (all strains combined) of all the mosquitoes recaptured, a significantly lower proportion of blood-fed mosquitoes were recovered in the experimental huts installed with ITS that were new [6%, OR: 0.02 (95% CI: 0.01–0.03), p-value < 0.0001] or aged [16%, OR: 0.09 (95% CI: 0.05–0.14), p-value < 0.0001] compared to huts installed with aged Olyset^®^ Plus net (46%), Table 2; Fig. 4. Almost all the strains had significantly lower proportions of blood-feeding in the huts installed with ITS (*p* < 0.0001), with the exception of *An. funestus* mosquitoes, that blood-fed in similar proportions in the huts installed with aged ITS and huts with aged Olyset^®^ Plus net [OR 0.63 (95% CI: 0.32–1.24), *p* = 0.182] (Table S6).

### Insecticide-treated screening prevents mosquito indoor entry

Installation of ITS in the experimental huts significantly reduced entry of all mosquito strains tested in this study, both new [median 0, Incidence Rate Ratio (IRR): 0.10 (95% CI: 0.08–0.13), p-value < 0.0001] and in aged condition [median 2, IRR: 0.25 (95% CI: 0.21–0.31), p-value < 0.0001] compared to aged Olyset^®^ Plus net with a median 13 mosquitoes caught indoors (Table 2; Fig. 4). Indoor densities were significantly reduced for new and aged ITS compared to aged Olyset^®^ Plus net among all the strains tested (Table S7, Fig. 4).

### Perceived adverse effects

None of the volunteers withdrew from the study due to any perceived adverse effect. Sneezing was the common symptom reported by the participants of the trial (Table S8).

### Quality checks cone bioassay and chemical analysis

Median temperature was 26.0 °C (Interquartile Range (IQR): 25.4–27.0) and relative humidity was 69.8% (IQR: 47.8–76.4) during the implementation of cone bioassays, meeting recommended bioassay temperature conditions, and approximately 25% of relative humidity recorded were out of range [30]. Cone bioassays conducted on three samples (measuring 25 cm x 25 cm) of ITS before artificial aging revealed complete knockdown (100%) after 60 min of exposure and minimum mortality of 73% at 24 h against *An. arabiensis*, *An. funestus* and *Ae. aegypti* mosquitoes (Table S9). After SFS experiment, the proportion of mortality from new ITS (67% vs. 23%), aged ITS (67% vs. 10%), and Olyset^®^ Plus net (93% vs. 15%) reduced substantially against all species tested (Table S9). Chemical analysis indicated low residual content of deltamethrin (27%) and PBO (8%) in aged ITS (Table S10).

### Simulated Epidemiological Potential of Insecticide-treated Screening

**Fig. 5:**
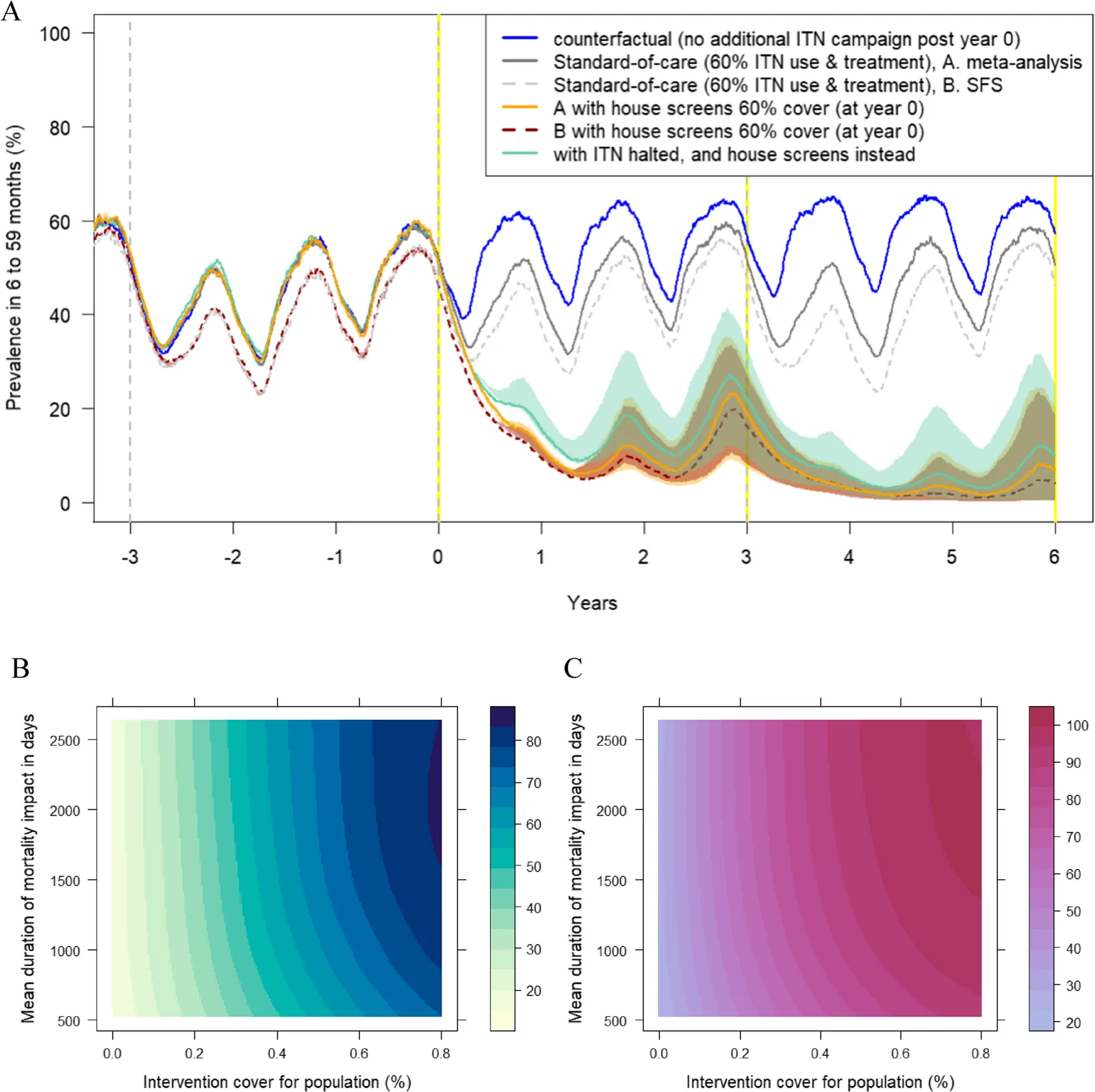
A transmission simulation exercise to explore the potential epidemiological impact of insecticide-treated screening (ITS). **A** A theoretical set of scenarios showing the microscopy-measured *P. falciparum* parasite prevalence in children of 6 to 59 months of age over time given implementation of the insecticide-treated bednet (ITN), and ITS, covering 60% of people on implementation. ITN distribution occurs every three years (vertical grey dashed lines). Net use and the potency of the insecticide both wane over time according to estimates from Sherrard-Smith et al. 2022 [57] (grey solid line), or the SFS data (grey dashed line). The effect of removing this ITN intervention from year 0 onward is indicated by the blue line (where the corresponding incidence outputs from this simulation are used as the respective counterfactuals for panels **B** and **C**). The impact of ITS used in addition to ITNs is shown by the solid orange line (with meta-analysis parameters for ITNs), and red line (with SFS data parameters for ITNs). The impact of ITS as an alternative to ITNs is shown as the green line. Uncertainty that is due to the upper and lower estimates shown in Fig. 5A is demonstrated by the corresponding polygons. All other model parameters are matched between runs. **B** The estimated relative reduction in mean prevalence in children of 6 to 59 months of age given deployment of an ITS with specified population cover and mean duration of mortality impact on mosquitoes, compared to the counterfactual simulation, over the three years (time 0 to 3 years in panel **A**). **C** The percentage reduction in all-age clinical incidence against the same counterfactual

The SFS data indicated that the pyrethroid-PBO ITN in the tested system (Supplementary Fig. 4) was working better than the average parameter estimates for the product derived from a meta-analysis review of experimental huts [57, 58], Table S2. This translates to an improved epidemiological impact simulated in the transmission model for the SFS-derived parameters, where the SFS-derived parameters are at the upper bound of the meta-analysis-derived parameter uncertainty shown in Fig. 5A. The reduction in mean prevalence in children of 6 to 59 months of age over three years, relative to a scenario simulating the cessation of mass ITNs distribution previously achieving 60% use by the population at implementation, that is achieved by the continuation of the pyrethroid PBO ITN campaign is 23% (21– [24]) for the SFS parameterisation, and 15% [3–21] for the meta-analysis parameters. The additional implementation (given SFS parameters) of the ITS product to cover 60% of the population in a comparable scenario reduces prevalence in the same age cohort by 76% [65–79] (and this is 71% [62–78] for the meta-analysis pyrethroid-PBO ITN parameters together with ITS). Used alone, the modelled impact from the ITS remains impressive, achieving a reduction in prevalence among children of 6 to 59 months of 65% [51–72] relative to no further ITN campaigns from year zero.

In the absence of empirical data, a sensitivity analysis using the transmission model investigated how different assumptions on the durability of the screens and its mortality-inducing impact could shift efficacy. This theoretical investigation suggests that increasing the durability of the mortality-inducing impact and increasing the cover of people within the population who are benefiting from the product is beneficial (Fig. 5B and C). Even when the mean duration of impact on mortality is low, between one and two years, achieving screening for 60% of the population may be able to reduce mean prevalence in children of 6 to 59 months of age over three years by 60% compared to no ITNs (Fig. 5B), and all age clinical incidence by over 80% (Fig. 5C).

## Discussion

The most impactful mode of action of vector control products is induced mortality of mosquito vectors [64, 65]. This reduces mosquito population size and mean age, so that fewer mosquitoes survive long enough for the pathogen they carry to mature to its infective stage. Mosquito population-wide effects of insecticides reduce the overall burden of disease among not only users but also non-users of the intervention living in the same community. In our study, the experimental huts installed with ITS killed more than half of the pyrethroid-resistant mosquitoes released, likely due to the use of deltamethrin with the addition of the synergist PBO, which increases the potency of pyrethroids by reducing detoxification [29]. The corresponding modelling exercise offers support that, as an additional vector control tool, treated screening is capable of impacting malaria prevalence. The concept of shielding entry points with insecticidal barriers has been shown to be successful in reducing the entomological inoculation rate and incidence of malaria in a trial of eave tubes that incorporated an insecticide [20]. ITS has an added advantage in that it can be applied to unimproved housing by wrapping the eaves and tacking simple screens over windows and areas of damage in mud-walled houses. Given the increasing resistance to pyrethroids in Africa [66] and the reduction in the number of IRS campaigns conducted by many malaria programmes [2], the use of non-pyrethroids on ITS for insecticide resistance management would be beneficial [67]. Whilst the insecticides on ITNs need to meet strict safety criteria and must not be soluble in water when washed, ITS may offer an alternative delivery method for a wider range of water-soluble insecticides (as screens are unlikely to be washed) and may have a toxicity profile not suitable for ITNs [5]. This increases the range of possible active ingredients available for controlling vector mosquitoes.

A similar proportion of mosquito mortality was observed between huts installed with aged ITS (51%) and aged Olyset^®^ Plus ITN (55%). Confirmatory cone bioassay and chemical testing indicated substantial loss of bioefficacy and loss of pyrethroid-PBO content after one year of ageing. The aged ITS was exposed to environmental conditions, including tropical temperatures, wind, and dirt. ITS on windows was additionally exposed to ultraviolet light from sunlight. Studies have shown that the degradation of insecticide in ITNs is principally due to ultraviolet light [68], and the long-term exposure of the ITS installed on windows to sunlight when installed during ageing might have contributed to the degradation of both the insecticide and synergist incorporated within it. While it was not clear if the ITS on eaves were exposed to direct sunlight, evaporation or blooming of deltamethrin into large crystals with lower bioavailability might be a major reason for their content loss. The Olyset^®^ Plus ITN used in this experiment was not exposed to direct sunlight as it was hung inside the experimental system, however maximum indoor temperature (47.4 °C) exceeded 32 °C tolerance for AI [30]. Loss in its potency was likely through evaporation and washing as has been reported elsewhere [69]. In our study, no holes were observed in the ITS after one year of installation in the animal house where the presence of animal feed is likely to encourage rodents. Similarly, the ITS was observed intact after four years in the Kenyan pilot study [28]. This may be due to the lower household interaction with ITS compared to ITN. Therefore, optimisation of the insecticide formulation is needed to retain insecticide content for the duration of the product’s life allowing the ITS to be a cost-effective method for controlling mosquito-borne diseases [70].

The modelling exercise performed here indicates the epidemiological potential of a house screening product that can also induce mosquito mortality. The year-old ITN performed better than the average effects we might have expected from a systematic review performed previously [58]. This might be due to the different process used to artificially age the ITN in our study (6 washes), compared to that in experimental hut trials (20 washes), or 72-hour mortality used in this study compared to 24-hour mortality conventionally used for AI with fast mode of action [71], or impacts in semi-field system (SFS) is often higher than reality or experimental hut trials [72]. Either way, the ITS outperformed the year-old ITN. Our like-for-like modelling comparison, however, assumes ITS is implemented overnight to the community at the 60% coverage level which is likely not possible, especially as the target population increases in size. The gain from the ITS may be more gradual therefore than depicted in Fig. 5.

The simulation exercise shows that the protective impact can be lasting even as mortality-inducing effects wane. An advantage of this product over ITNs is their capacity to protect all household members equitably. While the model does not target ITN use to any particular group (a limitation), studies have shown that teenagers are least likely to sleep under mosquito ITNs [73, 74], and social hierarchies sometimes lead to vulnerable household members being exposed through no access to ITNs [75]. Failing to protect the sleeping space leaves individuals at risk of infection, and even if individual risks of illness are low given acquired immunity, these individuals may act as reservoirs for malaria parasite transmission [76, 77]. The comparative analysis completed may be overestimating the initial impact, given the likely longer time required to implement a house screening intervention compared to ITNs that can be deployed almost simultaneously through various operational strategies. Nevertheless, the likely durability of screening will advantage those enjoying its benefits; even while mortality impacts wane, holes are less likely to be accrued than for interventions that are manipulated more often, like ITNs. The sensitivity analysis indicates that even with a relatively low cover of 40%, an impressive reduction in prevalence and cases may be achievable with a product that has a mean mortality-inducing duration of only one to two years (Fig. 5B, C). The modelling exercise raises the possibility of considering such a product as an alternative to ITNs where resources are not able to offer people both.

While the efficacy of the ITS varied by mosquito species the trends were consistent. The proportion of mortality of *Anopheles (An.) funestus* mosquitoes was approximately 13% lower in huts installed with aged ITS than aged Olyset^®^ Plus ITN. This may be due to the greater reduction in insecticide and synergist content, the insecticide resistance profile of the vector, and also its small size enabling it to pass more easily through the ITS. It is important to ensure that the ITS is able to kill *An. funestus* given its competence in transmission of the malaria parasite [48], and widespread distribution throughout sub-Saharan Africa. Investigations of alternative non-pyrethroid insecticides for treating ITS to target this species are essential.

The dengue vector *Aedes (Ae.) aegypti* mosquitoes were not killed in as high proportions as the malaria vectors, despite being susceptible to pyrethroids (Table S1). A similar observation was found in the laboratory testing of the ITS [53], which is attributed to the doubled release ratio of PBO to deltamethrin, therefore less deltamethrin was bioavailable to sufficiently impact susceptible *Ae. aegypti* given that PBO is a synergist. Similarly, the nuisance biter, *Culex (Cx). quinquefasciatus*, also demonstrated lower mortality, which could be explained by its high resistance mediated through multiple mechanisms [51]. However, both were still kept out of houses and prevented from blood-feeding. Relief from the bites of *Cx. quinquefasciatus* may increase the likelihood of adoption of ITS [78]. It is also possible that the wide-scale installation of ITS, even if designed for malaria control may have some impact on dengue transmission. Further work is required to evaluate this.

Although ITS lost the majority (> 70%) of its deltamethin and PBO content due to the high release rate technique, it was still able to prevent mosquito blood-feeding and reduce hut entry. This is consistent with studies that have investigated house modification and insecticide-treated eave nets [37, 38]. The physical barrier afforded by the intact netting and the remaining amount of active ingredient and synergist that could induce disorientation or prevent host-seeking could explain these results. Such ‘sub-lethal’ effects have the potential to also directly impact disease transmission [64, 72], and reduce mosquito fitness that may eventually lead to mosquito mortality [79].

The risks (high hut entry, high blood-feeding, and low mortality) of mosquitoes in the huts without any treatment were extremely high, stressing the need that all houses in the community should be given some form of intervention. Considering that the use-life of ITNs, the conventional malaria prevention tool [11] is often less than two years [80], and that their mass distributions are often conducted every three to four years [8], many households may be left unprotected in the community particularly at the later stages of ITN campaign cycles. This scenario may partially explain observed stagnant or rebound trends in malaria prevalence in some communities, as demonstrated in Zambia [81]. Deploying long-lasting ITS as a house modification tool when access to effective ITNs decline, and as communities wait for mass campaigns or top-up distributions, has the potential to cover that gap in sufficient protection.

The proportion of mosquitoes found was lower in the arms with higher mortality thus likely systematically underestimating the most efficacious treatment. This may be due to ants removing dead mosquitoes or simply that they are difficult to find despite the precautions taken to reduce ant entry (water canal, and grease) and improve findability (white linen floors) [79]. The association between recapture number and mortality at 72 h was supported by an estimated Pearson Correlation of -0.0641 (p-value 0.0037). Ants were indeed observed in the SFS in the first round of the tests and were subsequently controlled using four ant bait stations manufactured by Neudorff, Switzerland. Each station was placed in the corridor and in proximity to each chamber, therefore their presence was unlikely to have an impact on the efficacy of ITS. Ant control is an essential prerequisite for supporting a high proportion of mosquito recapture in SFS experiments, particularly if knockdown or mortality endpoints are to be assessed.

Further work on ITS is also required to ensure adverse environmental impacts are minimised and that no adverse effects on users are experienced. Our results on the latter are promising. Reported adverse effects, especially sneezing, were reported among all the hut sleepers, with events reported as more common among those who slept under ITS and ITN. This may indicate a response to the exposure to the pyrethroids and/or-PBO [30]. It was determined that the reported effects were tolerable, as none of the volunteers withdrew from the study as a result of the events. The next steps include consideration of any indirect environmental impacts from scaled use of the treated screen, such as mortality or morbidity effects on non-target species living in and around homes, and any leaching of microplastic or pesticide into soils, air or waterways. For any polyester or polyethylene product that includes insecticide, there are concerns for environmental and human health. We are unaware of any study reporting on adverse effects for humans who have had long-term exposure to bednets of this description, which represent a more acute risk given the proximity to an individual’s skin. There are growing concerns on the impact from this type of netting on fishing stocks, given the fine mesh and insecticide contamination risk from netting that is misused or repurposed as fishing equipment [82–84]. Were the ITS to be adopted at scale, we would need to consider how to mitigate against such challenges.

Standardised bioassays are often needed to assess efficacy of vector control tools before they can be recommended for public use [30, 31]. Enclosed SFS that allow mosquitoes within ambient conditions to free-fly can be used to estimate effects against more naturally behaving populations than laboratory bioassays. SFS studies have been used to assess insecticidal screens in other studies [32, 33, 37]. Furthermore, the potential for a high proportion of recaptures of mosquitoes that have encountered ITS in our SFS study allowed for robust measurements of relevant endpoints [72]. SFS bioassays use laboratory-reared mosquitoes of a set density, physiological status, and age, which removes some of the variability seen in field studies [85]. The use of laboratory-reared uninfected insects also removed the risk of occupants within the SFS being exposed to pathogens through the bites of mosquitoes, especially in areas of ongoing arbovirus transmission [86].

Products were naturally aged for only one year. Studies for over three years would have provided important information on their comparative efficacy to ITNs. Incorporating a new and unused Olyset^®^ Plus ITN as well as ITS + ITN would have provided a more comprehensive comparative efficacy of ITN to the new ITS and how ITS works alongside ITN. Furthermore, the ITS used in this study had been stored at IHI for over a year prior to ageing in sacs that did not prevent the insecticide and synergist loss. Consequently, some of the insecticide and synergist may have already evaporated. Even the ITS considered new might have experienced insecticide and synergist loss due to a similar year-long storage period. Therefore, the efficacy of ITS observed in this study may be an underestimate. We recommend future studies to be conducted with new netting (ITS and ITN) with a low storage period (< 6 months) and ITS aged (controlled as well as under real-life conditions) for a longer period, and with slow-release non-pyrethroid insecticides.

In the scenario analysis, we assumed relatively high indoor biting behaviours for mosquitoes given the estimates observed in sub-Saharan Africa [21], and this will indicate greater impact against transmissions than for areas where mosquitoes show greater exophilic behaviours – however, this is also true for other indoor interventions [87]. Our model parameters are based on a single SFS study. The estimated parameters for the ITN from our SFS data show impacts in the upper region of the meta-analysis for ITNs [57, 58], so caution is needed, given that the results may indicate a lower impact if repeated elsewhere. It may be useful to investigate ITS in another setting to estimate and compare its impact. Our assumption of mosquitoes having to pass the housing barrier to enter and leave a house after blood-feeding improved the benefits from the ITS compared to ITN. However, this seems reasonable as the barrier, and corresponding potential for mosquitoes to contact the mortality-inducing chemistries, remains in place as a mosquito tries to exit. Some may be able to leave via doors if other entry points, including eaves and windows, are screened, but we have not empirically tested this here and our assumption is that this is not occurring. Empirical studies in other settings are needed to confirm this and other assumptions to allow the model to be further validated using the SFS setting, such that the mechanism of action can be determined and quantified for modelling to be robust enough to extrapolate results more widely.

In summary, this study provides novel entomological and modelling evidence to support ITS as a promising intervention for malaria and arbovirus vector control that protects all members of the household without the need for regular compliance, and through insecticidal activity that may also induce community protection if applied at scale. Unlike many previous studies focused on ITNs or general housing improvements using untreated materials [9, 38], our work directly compares a new ITS product with the current standard of care pyrethroid-PBO-ITN using a rigorous semi-field evaluation to collect multiple entomological surrogates of disease control, across multiple vector species. The findings support ITS not only as an effective personal protection measure but also as a potential community-level intervention with durable, passive protection. Further evaluation in full field conditions and under operational delivery will help define its role within integrated vector management strategies in Tanzania and beyond. Such implementation trials should be planned based on these promising results to generate the data needed to inform long-term effectiveness, real-world implementation challenges (e.g., household refusals), insecticide resistance impacts, detailed epidemiological outcomes, durability, cost-effectiveness, specific vector species responses (especially resistant ones), and large-scale adoption barriers.

## Conclusion

This study shows that insecticide-treated screening (ITS) effectively reduces indoor mosquito entry, blood-feeding, and survival—including vectors of malaria, dengue, and nuisance species. Even after one year of natural aging, ITS performs comparably to 12-month field-used PBO-treated nets, the current standard in Tanzania. Its dual role as a physical barrier and targeted insecticide delivery platform, along with compatibility with a broader range of insecticides and suitability for unimproved housing, highlights ITS as a promising complementary or alternative vector control tool.

Our findings also highlight the public health potential of ITS as illustrated through mechanistic modelling, which projects sustained transmission reduction even with moderate coverage levels. By addressing current operational challenges such as ITN durability, suboptimal compliance, and the need for insecticide resistance management, ITS can fill critical protection gaps, particularly in households left vulnerable between ITN distribution cycles.

Given these findings, we recommend ITS be further evaluated for longer-term durability, sustained effectiveness, operational feasibility, large-scale adoption barriers and monitoring insecticide resistance across diverse vector species, and considered for inclusion in national malaria and arbovirus control strategies, particularly in regions with poor ITN usage, high resistance, and large populations living in unimproved housing.

## Supplementary Information

Supplementary Material 1. Table S1: The 24-hour mortality observed in the resistance testing. Table S2. Transmission model parameter ranges describing entomological impact from the pyrethroid-PBO bednets, and insecticide-treated screen as estimated from a systematic review of experimental hut trials (58) and modified for modelling and validated against RCTs (57), using the SFS data. The parameters shown are required to simulate the potential epidemiological impacts of these interventions. Table S3: Mosquito bionomics assumptions used in the transmission modelling. Table S4: Number of mosquitoes released and recaptured in the semi-field system experiment. Table S5 Mortality at 72 hours of mosquitoes exposed to insecticide-treated screening compared to a standard of care pyrethroid-PBO insecticide-treated bednet, Olyset® Plus in the semi-field system. Table S6 Blood-feeding of mosquitoes exposed to insecticide-treated screening compared to a standard of care pyrethroid-PBO insecticide-treated bednet, Olyset® Plus in the semi-field system. Table S7 Indoor density of mosquitoes exposed to insecticide-treated screening compared to a standard of care pyrethroid-PBO insecticide-treated bednet, Olyset® Plus in the semi-field system. Table S8 Adverse events reported by the sleepers in the SFS within the first four nights of sleeping. Table S9 Summary of quality check outcomes against laboratory-reared strains in Tanzania before and after SFS testing. Table S10 Insecticidal content of insecticide-treated screening before and after ageing. Table S11: Model parameters determined and reported to allow the simulation of the insecticide-treated benet or insecticide-treated screen interventions. Figure S1: Map of Tanzania showing the location of the semi-field system. Figure S2: Sample cutting for post-semi-field system experiment quality checks. Figure S3: A narrative schematic to summarise the key features of the malariasimulation transmission model. The left panel demonstrates the various states that humans can pass through during the simulation. The right panel outlines the dependency of the probable outcomes at the conclusion of each foraging cycle on the presence of interventions. Figure S4. Summary of semi-field trial data as arranged to represent the model parameters for mosquito repeating, feeding, and survival. Note: Boxplots show the median (solid central line) and 25th to 75th percentiles, boxed section, with 2.5th and 97.5th percentiles indicated by the whiskers. Data are overlaid for the different mosquito species tested: A pyrethroid-resistant strain of Anopheles arabiensis, (open circles) and An. funestus (closed circles); Susceptible Aedes aegypti (asterisks) and; pyrethroid-resistant Culex quinquefasciatus (open squares). See Methods for how data estimates are derived from raw data for ITNs or ITS. ITNs are shown in blue, new screens in orange, and year-aged screens in red. Figure S5. A schematic showing the modelled decision tree pathways for mosquito repetition, biting, and survival, in a household with a housing screen (see subscript h) and where people may be sleeping under an insecticide-treated bednet (see subscript n). We assume that a proportion of bites are taken outside (blue background; where there are no protective interventions), a proportion are attempted indoors but not on sleeping humans (green background; where they may encounter an insecticide-treated screen resulting in deterrence r h , killing d h or survival and a successful bite s h) and a proportion are attempted on a sleeping human in bed (yellow background; where in addition to ITS, they may additionally encounter an ITN, which again may result in deterrence r n, killing before they are able to bite d n, or successful biting s n ). Following a successful bite or repulsion by an ITN, a mosquito then must attempt to exit the house, passing again through the house screen, where she may again be deterred, killed, or pass through safely having fed. This time, mosquitoes are deterred back into the house (assuming ITS offers complete screening), where we make the assumption that they will eventually die. Bites occur at the positions on arrows marked with a red line. Symbol φ I: Proportion in mosquitoes attempting to bite humans indoors in the absence of interventions; φ B: Proportion of mosquitoes attempting to bite humans in bed in the absence of interventions.

## Data Availability

No datasets were generated or analysed during the current study.

## Abbreviations

*ACT*: *Artemisinin combination therapy*
*Ae*: *Aedes*
AI: Active Ingredients
*An*: *Anopheles*
CI: Confidence Interval
cm: Centimetre
*Cx*: *Culex*
dh: Degree of hardness
g: grams
HPLC: High performance liquid chromatography
IQR: Interquartile range
IRR: Incidence rate ratio
IRS: Indoor residual spraying
ITNs: Insecticide treated bednets
ITS: Insecticide treated screening
Kg: kilogram
M: Metres
mg: milligram
mm: Millimetre
M72: Mortality at 72 hours
OR: Odds ratio
PBO: Piperonyl-butoxide
PPE: Personal protective equipment
R: Range
RH: Relative humidity
SFS: Semi-field system
WHO: World health organization
°C: Degrees centigrade
