# Supplementary material for: House modifications using insecticide-treated screening of eave and window as a vector control tool: evidence from a semi-field system in Tanzania and simulated epidemiological impact
Source: BMC Public Health. 2026 Jul 17;26:3249. doi: 10.1186/s12889-026-28554-z (PMC13741755; doi:10.1186/s12889-026-28554-z)
Supplement: Supplementary file 1 [file 12889_2026_28554_MOESM1_ESM.zip › 12889_2026_28554_MOESM13_ESM.docx]

| Type of Adverse Event | NO TREATMENT | AGED OLYSET® PLUS ITN | AGED ITS | NEW ITS |
| --- | --- | --- | --- | --- |
|  | N | N | N | N |
| Skin Itching | 0 | 0 | 0 | 0 |
| Facial Burning | 0 | 0 | 0 | 1 |
| Sneezing | 1 | 1 | 3 | 3 |
| Running Nose | 0 | 0 | 0 | 0 |
| Headache | 0 | 0 | 0 | 1 |
| Nausea | 0 | 0 | 0 | 0 |
| Eye Irritation | 1 | 0 | 2 | 0 |
| Tears from eyes | 0 | 0 | 0 | 0 |
| Bad Smell | 0 | 0 | 0 | 1 |
| TOTAL | **2** | **1** | **5** | **6** |

The total observation is 16, that is, 4 people per night for the first 4 nights of the experiment
