# Supplementary material for: House modifications using insecticide-treated screening of eave and window as a vector control tool: evidence from a semi-field system in Tanzania and simulated epidemiological impact
Source: BMC Public Health. 2026 Jul 17;26:3249. doi: 10.1186/s12889-026-28554-z (PMC13741755; doi:10.1186/s12889-026-28554-z)
Supplement: Supplementary file 1 [file 12889_2026_28554_MOESM1_ESM.zip › 12889_2026_28554_MOESM16_ESM.docx]

| Parameter | Description | Source from Supplementary Material 1 |
| --- | --- | --- |
| Superscript *v* | A parameter that can have a distinct value for each different mosquito vector species simulated. |  |
| Subscript *i* | A parameter that can be specific to each individual human *i* simulated. |  |
| *y* | The probability of a mosquito biting during a single feeding attempt (see Figure S3). | Griffin et al. 2010 (6) |
| *w* | The probability of a mosquito biting and surviving during a single feeding attempt. | Griffin et al. 2010 (6) |
| *z* | The probability that a mosquito will be forced to repeat her foraging cycle during a single feeding attempt. | Griffin et al. 2010 (6) |
| $Q_{0}^{v}$ | The proportion of blood meals that are taken on humans, this is distinct for species *v.* | Killeen et al. 2017 (3) |
| *Y, W, Z* | Probability *y, w,* and *z* are dependent on the interventions present. Capital *Y* indicated the summed probabilities across the different interventions included (the use of insecticide-treated nets (ITNs), the use of insecticide-treated screens (ITS), or humans benefitting from neither, or both interventions). | Griffin et al. 2010 (6) |
| π*_i_* | the proportion of bites that a person *i* receives, without interventions present. | Griffin et al. 2010 (6) |
| *φ_I_* | A vector species-specific parameter that determines the proportion of bites taken indoors in the absence of interventions, that contributes to the estimate of bites that can be prevented by the ITS. | Sherrard-Smith et al. 2019 |
| *Φ_B_* | A vector species-specific parameter that determines the proportion of bites taken in bed in the absence of interventions, that contributes to the estimate of bites that can be prevented by the ITS, ITN, or both. | Sherrard-Smith et al. 2019 (5) |
| *r_n0_* | Deterrence outcome from pyrethroid-PBO ITN working optimally immediately after deployment. | This work, and a meta-analysis Sherrard-Smith et al. 2022 (9) |
| *d_n0_* | Mortality-inducing effect from pyrethroid-PBO ITN working optimally. | This work, and a meta-analysis Sherrard-Smith et al. 2022 (9) |
| *γ_n_* | Inferred mean duration of mortality impact from pyrethroid-PBO ITN when working optimally. | This work, and a meta-analysis Sherrard-Smith et al. 2022 (9) |
| *r_h0_* | Deterrence outcome from ITS working optimally immediately after deployment. | This work |
| *d_h0_* | Mortality inducing from ITS working optimally immediately after deployment (SFS data). | This work |
| *γ_h_* | Mean duration of mortality effect from pyrethroid-PBO ITN when working optimally, inferred from SFS data | This work |
