# Supplementary material for: House modifications using insecticide-treated screening of eave and window as a vector control tool: evidence from a semi-field system in Tanzania and simulated epidemiological impact
Source: BMC Public Health. 2026 Jul 17;26:3249. doi: 10.1186/s12889-026-28554-z (PMC13741755; doi:10.1186/s12889-026-28554-z)
Supplement: Supplementary file 1 [file 12889_2026_28554_MOESM1_ESM.zip › 12889_2026_28554_MOESM17_ESM.docx]

**The *malariasimulation* transmission model**

*Malariasimulation* is an open source, open access, mathematical model that tracks the transmission of *falciparum* malaria between humans and mosquitoes. Comprehensive details are found here: [mrc-ide/malariasimulation: The malaria model (github.com)](https://github.com/mrc-ide/malariasimulation)

A narrative of the model is presented in Figure S3. Here, the main elements of the transmission model are outlined. Humans are born susceptible to infection and have some maternally inherited immunity that wanes after six months from birth. An infectious bite might cause infection depending on the levels of immunity (maternal, pre-erythrocytic, and immunity against clinical disease), the levels of infectivity of the mosquitoes, the biting rate of mosquitoes, and the population size of mosquitoes. An infective bite causes the host to become clinically ill or develop asymptomatic patent infection, at rates depending on immunity. Clinically ill people can be successfully treated and receive partial protection for a limited time depending on the drug administered. Patients move to the asymptomatic sub-patent infection compartment depending on parasite densities and can eventually clear parasites to return to the susceptible state.

It is the biting rate that is important for our model adjustments. The mosquito component is outlined in more detail below, but essentially, adult female mosquitoes are tracked through their gonotrophic and foraging cycles and the assumptions here determine the regularity of bites on susceptible people. At the conclusion of the cycle, there is some probability that a bite occurred on a person, a probability that the mosquito successfully bites and survives and a probability that the mosquito was forced to repeat her foraging attempt, delaying the subsequent blood-feed. In the absence of interventions, these probabilities are 1, 1 and 0 respectively. In the supplementary information below, we outline how these probabilities are altered, and the key aspects of the transmission model adaptions that enable the simulation of the insecticide-treated screening (ITS). For completion, we also outline how insecticide-treated nets (ITNs) alter the probable outcomes of foraging cycles.

**Model changes introduced in this manuscript**

The vector model is compartmental and deterministic, tracking different species of mosquito from eggs through to adults. Only adult females are tracked after emergence from pupae, and the sex ratio at emergence is assumed 50:50 (1). The mosquito carrying capacity is assumed to track rainfall patterns locally (2). Adult mosquitoes seek hosts after emerging, can oviposit after successfully feeding, and their mortality rate, prior to interventions are assumed as indicated in Table S3 for the species included in our simulation exercise (*Anopheles (An.) arabiensis* and *An. funestus*). The proportion of bites taken on humans is estimated following estimates provided by Killeen et al. 2017 and Massey et al. 2016 (3, 4). The probability that bites are attempted indoors or in bed in the absence of indoor interventions is estimated (5). Life expectancy, foraging, and blood-feeding rates are taken from Griffin et al. (2010) (6). Table S11 shows parameters and descriptions.

In the transmission model, the probability of a mosquito of a given species *v* biting host *i* during a single blood-feeding attempt is defined as $y_{i}^{v}$; the probability of the mosquito biting and surviving the feeding attempt is $w_{i}^{v}$, and the probability that she is deterred without feeding is $z_{i}^{v}$. Given these probabilities exclude natural vector mortality with no protection, these probabilities are $y_{i}^{v}=w_{i}^{v}=1$ and $z_{i}^{v}=0$. Not all mosquitoes feed successfully when entering a house so$y_{i}^{v}$,$w_{i}^{v}$ and $z_{i}^{v}$must take account of the potential repeating behaviour of female mosquitoes foraging prior to introducing insecticides.

Following Le Menach et al. (2007) (7) and Griffin et al. (2010) (6), we add the potential impact from barriers to the model equations tracking the probable outcomes of a female vector feeding attempt through her oviposition cycle. During a single feeding attempt on either animals or humans, a mosquito will feed successfully with probability $W^{v}$ such that:

$W^{v}=1-Q_{0}^{v}+Q_{0}^{v}\sum_{i} \pi_{i}w_{i}^{v}$, [Equation S1.1]

Where the last term in the equation is the probability that she will be deterred without feeding ($Z^{v}$):

$Z^{v}=Q_{0}^{v}\sum_{i} \pi_{i}z_{i}^{v}$, [Equation S1.2]

Here, $Q_{0}^{v}$ represents anthropophagy which is defined in the model as the proportion of bites taken on humans by species $v$ when interventions are absent (Table S2). Similarly, $\pi_{i}$ denotes the proportion of bites that a person *i* receives, without interventions present. Protection due to insecticide-treated screening (ITS) and insecticide-treated nets (ITNs), both depend on the proportion of bites that are taken when a person is protected by these interventions, and this depends on human sleeping patterns and mosquito activity. In the model, we define the proportion of bites taken indoors as *φ_I_* and the proportion of bites taken in bed as *φ_B._* Assumed values for these probabilities are shown in Table S3 for *An. arabiensis* and *An. funestus* mosquitoes.

When a mosquito attempts to enter a house, she can do so successfully (feeding and surviving, *s*), be killed (*d*), or be forced to repeat her search (*r*). Here, we denote the success, death, or repetition due to house screening as *s_h_, d_h,_* and *r_h_* respectively, following the notation from (Griffin et al 2010) (6), [a superscript *v* could be included for species but we leave this out for simplicity]. Similarly, *s_n_, d_n,_* and *r_n_* show the corresponding outcomes with ITNs. Figure S4 demonstrates this pathway as depicted previously for ITNs by Griffin et al. (2010) (6) with the addition of some form of house screening which induces a mortality effect (*d_h_*), and the removal of IRS impacts consistent with the SFS study design. The ITS is the first commodity that may be experienced by the indoor biting mosquito, where we assume that she may be deterred, killed, or fed successfully. A mosquito that attempts to bite humans in bed may encounter an ITN where she may again be deterred, killed, or fed successfully. If she survives (regardless of whether she was deterred or was able to feed), she must then exit the house passing the house screen (assuming that the screening completely covers all entry points) a second time, where she may again be deterred, killed, or exit the house. We assume that if she is deterred during her second encounter with the house screen, she moves back into the house space, where she will be killed (assuming 100% kill from the screen) given she will encounter ITS in her attempt to exit.

The r*_h_*, *d_h,_* and r*_h_* terms are therefore included at both stages of the pathway, entering and exiting the building.

In the absence of ITS, both *r_h_* and *d_h ­­_*are set to 0, and *s_h_* is set to 1, which means that mosquitoes cannot be trapped inside the house. Similarly, in the absence of ITNs, *r_n_* and *d_n_* are 0, while *s_n_* is 1.

Given this structure, it is possible to estimate the probability of a mosquito of a given species being deterred, biting, or successfully feeding depending on the combination of interventions in action. We adjust the (Griffin et al 2010) (6) probabilities to include the effects from house screening, (while removing the impact of IRS), and define the probability of surviving a successful feeding (*w_i_*) as:

$1-\varphi_{I}+\left( \varphi_{B}s_{n}s_{h}^{2} \right)+(\varphi_{I}-\varphi_{B})s_{h}^{2}$ [Equation S1.3]

Similarly, we introduce the potential impacts of ITS to the probability of biting (*y_i_*) such that:

$1-\varphi_{I}+\left( \varphi_{B}s_{n}s_{h} \right)+(\varphi_{I}-\varphi_{B})s_{h}$ [Equation S1.4]

And the probability of repeating (*z_i_*) as:

$\varphi_{B}{s_{h}}^{2}r_{n}+\varphi_{I}r_{h}$	[Equation S1.5]

This mechanism allows us to set a fraction of a population to receive some form of protection from the housing adaptations, to simulate that the household changes reduce the entrance of mosquitoes by a defined quantity and that some mosquitoes will be killed on contact with the screens.

Unless otherwise stated, all parameter estimates can be found here: [malariasimulation/R/parameters.R at master · mrc-ide/malariasimulation (github.com)](https://github.com/mrc-ide/malariasimulation/blob/master/R/parameters.R), or here: [malariasimulation/R/vector_parameters.R at master · mrc-ide/malariasimulation (github.com)](https://github.com/mrc-ide/malariasimulation/blob/master/R/vector_parameters.R)

**Model parameterisation**

The SFS data (see Supplementary Material 2) is used here to parameterise the ITN and ITS impacts within the model simulations. However, the aged ITN provides a measure of the impact at one year and to appropriately simulate the effects, we need to translate this to an optimal impact i.e. the impact that would be afforded on first deploying new ITNs to the community.

To choose representative model parameters for the ITS, only the recaptured mosquitoes are considered. For each data point (daily count of mosquitoes given species and treatment), we determine the parameter estimates following the pathways in Figure S5. This means that, the mosquitoes recovered in the SFS data that are outside of the hut, fed and alive, out of the total recaptured, represent those who have passed into and out of the house in the screening treatment group. These are thus, *s_h_^2^*, so we must take the square-root to estimate *s_h_* for the model parameter (Figure S5). The corresponding proportion of mosquitoes who have been forced to repeat their feeding attempt *r_h_* can be estimated by the mosquitoes counted outside and alive, but unfed out of the total recaptured. Then, *d_h_ = 1 – s_h_ - r_h_*.

In the one-year-old ITN treatment, the proportion of mosquitoes that were recovered dead (either indoors or outdoors and either fed or unfed) represents the parameter *d_n,t = 365 days_*, where *t* can indicate optimal performance for newly applied netting (*t* = 0), or one-year-old performance (*t* = 365 days) as appropriate*_._* The parameter *r_n,t = 365 days_* is calculated as the proportion of mosquitoes who are recaptured outside and alive out of the total recaptured during the SFS. The corresponding estimate for *s_n,t = 365_* is calculated as 1 – *r_n_,_t = 365 days_* – *d_n,t = 365 days_*.

The full durability of ITNs is unknown, so – for modelling purposes –an assumption is made on how rapidly these impacts decay following the mass campaign. Previously, a mean duration of mortality-inducing impact has been estimated by Griffin et al. (2010) [69] from Mahama et al. (2007) [73] for pyrethroid-only ITNs using washed ITNs, and the same logic applied for pyrethroid-PBO ITNs (8). This assumes that the killing effect from ITNs will decrease at a constant rate *γ_n._*.

The impacts from implementing ITNs are projected to changes so that:

$$r_{n}=\left( r_{n0}-r_{nm} \right){exp}^{-t\gamma_{n}}+r_{nm}$$

$$d_{n}=d_{n0}{exp}^{-t\gamma_{n}}$$

$$s_{n}=1-r_{n}-d_{n}$$

Applying these rates of decay to the data from the SFS, and back-calculating for the pyrethroid-PBO ITN entomological impact at time zero returns higher mortality estimates than a meta-analysis that was conducted previously to estimate ITN durability across Africa (9, 10). Both the meta-analysis parameter estimates and those estimated from the empirical data are simulated and values are reported in Table S2.

In the absence of further information on the durability of the ITS, we explore a reasonable range in the mean duration of the product mortality-inducing impact that would return mortality estimates after 365 days within the range of the SFS results. A minimum deterrence impact is set for both ITNs (*r_mn_*) and ITS (*r_mh_*). For ITNs, this estimate is assumed to be 0.24 (6). For the ITS, we cap this at 0.25 indicating a 25% reduction in entry assuming it is unlikely that the barrier will be lost even as insecticide potency wanes and it is assumed to be less likely that holes will accumulate as rapidly in the screen netting than the ITN netting. We mirror the structure for waning efficacy from ITNs into the mechanism of action for ITS such that:

$$r_{h}=\left( r_{h0}-r_{hm} \right){exp}^{-t\gamma_{h}}+r_{hm}$$

$$d_{h}=d_{h0}{exp}^{-t\gamma_{h}}$$

$$s_{h}=1-r_{h}-d_{h}$$

In addition, we include simulations that use pyrethroid-PBO ITNs parameters developed via the statistical framework and validated against gold-standard randomised control trial data for testing the epidemiological impact of these interventions (9). In the review work preceding the validation exercise (10), associations between entomological impact and the level of phenotypic resistance in *Anopheles* populations were statistically determined allowing the resistance-level specific parameterisation of impacts on mosquitoes from ITNs within the transmission model. Values aligning with the resistance status of the species *An. arabiensis* and *An. funestus* tested within the SFS are used here for the model simulation exercise (Table S2).

**References**

1.	White MT, Griffin JT, Churcher TS, Ferguson NM, Basanez MG, Ghani AC. Modelling the impact of vector control interventions on *Anopheles gambiae* population dynamics. Parasit Vectors. 2011;4:153.

2.	Winskill P, Slater HC, Griffin JT, Ghani AC, Walker PGT. The US President's Malaria Initiative, *Plasmodium falciparum* transmission and mortality: A modelling study. PLoS Med. 2017;14(11):e1002448.

3.	Killeen GF, Kiware SS, Okumu FO, Sinka ME, Moyes CL, Massey NC, et al. Going beyond personal protection against mosquito bites to eliminate malaria transmission: population suppression of malaria vectors that exploit both human and animal blood. BMJ Glob Health. 2017;2(2):e000198.

4.	Massey NC, Garrod G, Wiebe A, Henry AJ, Huang Z, Moyes CL, et al. A global bionomic database for the dominant vectors of human malaria. Sci Data. 2016;3:160014.

5.	Sherrard-Smith E, Skarp JE, Beale AD, Fornadel C, Norris LC, Moore SJ, et al. Mosquito feeding behavior and how it influences residual malaria transmission across Africa. Proc Natl Acad Sci U S A. 2019;116(30):15086-95.

6.	Griffin JT, Hollingsworth TD, Okell LC, Churcher TS, White M, Hinsley W, et al. Reducing *Plasmodium falciparum* malaria transmission in Africa: a model-based evaluation of intervention strategies. PLoS Med. 2010;7(8).

7.	Le Menach A, Takala S, McKenzie FE, Perisse A, Harris A, Flahault A, et al. An elaborated feeding cycle model for reductions in vectorial capacity of night-biting mosquitoes by insecticide-treated nets. Malar J. 2007;6:10.

8.	Churcher TS, Lissenden N, Griffin JT, Worrall E, Ranson H. The impact of pyrethroid resistance on the efficacy and effectiveness of bednets for malaria control in Africa. Elife. 2016;5.

9.	Sherrard-Smith E, Ngufor C, Sanou A, Guelbeogo MW, N'Guessan R, Elobolobo E, et al. Inferring the epidemiological benefit of indoor vector control interventions against malaria from mosquito data. Nat Commun. 2022;13(1):3862.

10.	Nash RK, Lambert B, NʼGuessan R, Ngufor C, Rowland M, Oxborough R, et al. Systematic review of the entomological impact of insecticide-treated nets evaluated using experimental hut trials in Africa. Current Research in Parasitology & Vector-Borne Diseases. 2021;1.
