# Supplementary material for: House modifications using insecticide-treated screening of eave and window as a vector control tool: evidence from a semi-field system in Tanzania and simulated epidemiological impact
Source: BMC Public Health. 2026 Jul 17;26:3249. doi: 10.1186/s12889-026-28554-z (PMC13741755; doi:10.1186/s12889-026-28554-z)
Supplement: Supplementary file 1 [file 12889_2026_28554_MOESM1_ESM.zip › 12889_2026_28554_MOESM6_ESM.docx]

| Test systems | Origin | Year of colonisation | established in Bagamoyo | Testing period | Permethrin (0.75%) | Deltamethrin (0.05%) | Alpha-cypermethrin (0.05%) | Lambda-cyhalothorin (0.05%) | Pirimiphos methyl (0.25%) | PBO (4%) | Permethrin (0.75%) + PBO (4%) | Deltamethrin (0.05%) + PBO (4%) | Alpha-cypermethrin (0.05%) + PBO (4%) | Lambda-cyhalothorin (0.05%) + PBO (4%) |
| --- | --- | --- | --- | --- | --- | --- | --- | --- | --- | --- | --- | --- | --- | --- |
| *Anopheles arabiensis*  (Kingani) | Kilombero, Tanzania | 2005 | 2011 | April 2021 | 3% | 46% | 12% | 22% | 100% | 0% | 98% | 100% | 100% | 100% |
|  |  |  |  | March 2022 | 11% | 22% | 17% | 21% | 98% | 5% | 99% | 100% | 100% | 100% |
| *Anopheles funestus*  (FUMOZ) | NICD South Africa | 2000 | 2021 | Feb 2021 | 61% | 34% | 10% | 17% | 100% | 2% | 100% | 100% | 100% | 100% |
|  |  |  |  | March 2022 | 60% | 78% | 72% | 66% | 100% | 6% | 100% | 100% | 100% | 100% |
| *Aedes aegypti*  (Bagamoyo) | Bagamoyo, Tanzania | 2015 | 2015 | Aug 2021 | 100% | 100% | 100% | 100% | 100% | - | - | - | - | - |
|  |  |  |  | March 2022 | 100% | 100% | 100% | 100% | 97% | 3% | - | - | - | - |
| *Culex quinquefasciatus*  (Bagamoyo) | Bagamoyo, Tanzania | 2019 | 2019 | March 2021 | 10% | 39% | 17% | 29% | 90% | 2% | 65% | 77% | 55% | 81% |
|  |  |  |  | March 2022 | 14% | 7% | 11% | 32% | 45% | 1% | 52% | 48% | 44% | 58% |

- Denotes that tests were not conducted at that time.
