# Supplementary material for: House modifications using insecticide-treated screening of eave and window as a vector control tool: evidence from a semi-field system in Tanzania and simulated epidemiological impact
Source: BMC Public Health. 2026 Jul 17;26:3249. doi: 10.1186/s12889-026-28554-z (PMC13741755; doi:10.1186/s12889-026-28554-z)
Supplement: Supplementary file 1 [file 12889_2026_28554_MOESM1_ESM.zip › 12889_2026_28554_MOESM7_ESM.docx]

| Parameter value and description | | Parameter ranges simulated | |
| --- | --- | --- | --- |
|  | Description | *An. arabiensis* outcomes: assuming 3 - 46 % of mosquitoes are killed on exposure to discriminating dose of pyrethroid at tube bioassay testing | *An. funestus* outcomes: assuming 10 - 78 % of mosquitoes are killed exposure to discriminating dose of pyrethroid at tube bioassay testing |
| Pyrethroid PBO insecticide-treated nets (ITNs) meta-analysis parameters | | Median, 10^th^ - 90^th^ percentiles | |
| *r_n0_* | Deterrence outcome from pyrethroid-PBO ITNs working optimally immediately after deployment | 0.500 (0.567 – 0.471) | 0.467 (0.551 – 0.444) |
| *d_n0_* | Mortality inducing effect from pyrethroid-PBO ITNs working optimally immediately after deployment | 0.454 (0.261 – 0.504) | 0.500 (0.365 – 0.541) |
| *γ_n_* | Mean duration of mortality effect from pyrethroid-PBO ITNs when working optimally | 808 (378 – 998) days | 995 (582 – 1277) days |
| Pyrethroid PBO insecticide-treated net (ITN) semi-field system estimated parameters | | Median, 25^th^ – 75^th^ percentiles | |
| *r_n0_* | Deterrence outcome from pyrethroid-PBO ITN working optimally immediately after deployment | 0.28 (0.46 – 0.24) | 0.27 (0.34 – 0.24) |
| *d_n0_* | Mortality-inducing effect from pyrethroid-PBO ITN working optimally | 0.57 (0.48 – 0.57) | 0.72 (0.63 – 0.75) |
| *γ_n_* | Inferred mean duration of mortality impact from pyrethroid-PBO ITN when working optimally | 1392 (1016 – 1472) days | 1419 (1257 –  1472) days |
| Insecticide-treated Screening (ITS) | | Median, 25^th^ – 75^th^ percentiles | |
| *r_h0_* | Deterrence outcome from ITS working optimally immediately after deployment | 0.13 (0.04 – 0.25) | 0.04 (0.00 – 0.13) |
| *d_h0_* | Mortality inducing from ITS working optimally immediately after deployment (SFS data) | 0.72 (0.59 – 0.82) | 0.91 (0.80 – 0.95) |
| *γ_h_* | Mean duration of mortality effect from pyrethroid-PBO ITS when working optimally, inferred from SFS data | 774 (467 – 1795) days | 1284 (774 – 2101) days |
